# The Efficacy and Safety of Dexamethasone Intravitreal Implant for Diabetic Macular Edema and Macular Edema Secondary to Retinal Vein Occlusion: A Meta-Analysis of Randomized Controlled Trials

**DOI:** 10.1155/2022/4007002

**Published:** 2022-08-09

**Authors:** Li Xiaodong, Xie Xuejun

**Affiliations:** ^1^Chengdu University of Traditional Chinese Medicine, Chengdu 610075, Sichuan, China; ^2^Department of Ophthalmology, Affiliated Hospital of Chengdu University of Traditional Chinese Medicine, Chengdu 610072, Sichuan, China

## Abstract

**Purpose:**

The purpose of this meta-analysis was to evaluate the efficacy and safety of dexamethasone intravitreal implant (DEX) for the treatment of diabetic macular edema (DME) with retinal vein occlusion secondary to macular edema (RVO-ME).

**Materials and Methods:**

Relevant databases were searched to include randomized controlled trials (RCTs) evaluating DEX for DME and RVO-ME. The search was conducted until March 2022. Meta-analysis was performed using Rev Man 5.4.1 software after screening the literature by inclusion and exclusion criteria, extracting information, and evaluating the methodological quality of the included studies.

**Results:**

The study showed that DEX treatment of RVO-ME was associated with an improvement in best corrected visual acuity (BCVA) (MD = −9.08, 95% CI: −10.89–7.27, *P* < 0.00001) and central retinal thickness (CRT) (MD = 93.47, 95% CI: 28.55–159.39, *P*=0.005). DEX treatment of DME was significantly better than anti-VEGF treatment in terms of CRT reduction (MD = −72.35, 95% CI: −115.0–29.69, *P*=0.0009). The safety study showed that the risk of cataract from RVO-ME (OR = 5.06, 95% CI: 1.96 to 13.06, *P*=0.0008) and the incidence of high intraocular pressure (OR = 6.67, 95% CI: 3.46 to 12.86, *P* < 0.00001) were significantly higher with DEX than with anti-VEGF therapy. The risk of cataract from DME (OR = 4.70, 95% CI: 2.10 to 10.54, *P*=0.00022) was significantly higher with DEX than with anti-VEGF therapy (OR = 4.70, 95% CI: 2.10 to 10.54, *P*=0.0002). The incidence of high IOP (OR = 13.77, 95% CI: 4.96 to 38.18, *P* < 0.00001) was significantly higher with DEX than with anti-VEGF therapy.

**Conclusions:**

In patients with DME and RVO-ME, DEX was more efficacious but slightly less safe than anti-VEGF therapy.

## 1. Introduction

According to the 10th edition of the IDF Diabetes Atlas, the global prevalence of diabetes in people aged 20–79 years was estimated to be 10.5% (536.6 million people) in 2021, rising to 12.2% (783.2 million people) by 2045 [1]. DME is one of the most common and serious complications of DR, and RVO is the second most prevalent retinal vascular ischemic lesion after DR, with BRVO being more common than CRVO in clinical practice [2]. DME and RVO-ME are two common clinical complications of fundus disease that are prone to recurrent episodes, leading to vision loss and even blindness in patients. A recent real-world study of 25,658 patients reported that more than 2/3 of patients with DME had visual acuity below 0.5, which severely affected their visual health [3]. The main pathological changes of DME and RVO in the macula are the release of various inflammatory cytokines such as VEGF due to ischemia and hypoxia in retinal tissue, which can cause the loss of vision by disrupting the blood-retinal barrier (BRB) and promoting neovascularization. The formation of intraretinal and subretinal fluid accumulation causes ME in the fundus, and long-term repeated ME leads to irreversible damage to retinal structures, resulting in permanent low vision and even blindness [4]; therefore, timely detection and treatment of ME are extremely critical. Anti-VEGF drugs (e.g., razumab, bevacizumab, abciximab, and compazepam) and corticosteroids (e.g., dexamethasone intravitreal extended-release implant and fludrocortisone intravitreal extended-release implant) have become the first-line treatment options for DME and RVO-ME in recent years. DEX is a synthetic corticosteroid that inhibits inflammation and fibrovascular proliferation, enhances cell adhesion in the endothelium and retinal pigment epithelium (RPE), reduces the release of various damaging chemokines, and maintains the integrity of the BRB [5]; therefore, in 2014, DEX was officially approved by the FDA for use in secondary ME. Therefore, this study used a meta-analysis to conduct a comprehensive systematic evaluation of the safety and efficacy of DEX for the treatment of DME and RVO-ME, with the aim of providing evidence-based medical evidence for clinical decision-making and postmarketing risk management of drugs.

## 2. Materials and Methods

### 2.1. Literature Inclusion and Exclusion Criteria

#### 2.1.1. Inclusion Criteria

Inclusion criteria were as follows: (A) The study type was RCT. (B) Study subjects were patients aged≥ 18 years with a definite diagnosis of DME or RVO-ME. (C) Patients in the trial group used dexamethasone intravitreal implants and patients in the control group used anti-VEGF analogues or sham injections. (D) Effectiveness outcomes were improvement in BCVA and degree of reduction in CST/CRT. Safety outcomes were adverse drug reactions. The safety outcomes were incidence of adverse drug reactions, cataract occurrence or exacerbation, high intraocular pressure, subconjunctival hemorrhage, etc. (E) The language was English. (F) The dosing period and follow-up ≥6 months.

#### 2.1.2. Exclusion Criteria

Exclusion criteria were as follows: (A) duplicate publications; (B) articles that included only the most recent findings from the same cohort study; (C) papers that could not be extracted, transformed, or obtained data such as reviews, meta-analyses, or case studies.

### 2.2. Literature Search Strategy

The Cochrane Library, PubMed, Embase, Web of Science, and Clinical Trials. gov were searched until March 2022. Subject search terms were as follows: dexamethasone intravitreal implant; macular edema; retinal vein occlusion; diabetic retinopathy; and randomized controlled trial; and free search terms are listed in the accompanying table.

#### 2.2.1. Literature Inclusion and Data Extraction

The literature screening was completed independently by 2 investigators, and after excluding studies that clearly did not meet the inclusion criteria, the abstracts and full texts were further read to determine whether the inclusion criteria were met. Information from the included literature was extracted and cross-checked. In case of disagreement, they discussed and reached consensus with the 3rd investigator. The extracted information included the following: (A) basic information of RCTs and baseline conditions of patients in the trial and control groups; (B) interventions, outcome indicators, lost visits, and treatment; (C) indicators of study quality, including whether the randomization method was correct, whether allocation concealment was achieved, whether blinding was used, whether there were lost visits and withdrawals, whether there was selective reporting bias, and whether there were other biases.

#### 2.2.2. Data Extraction and Quality Assessment

The methodological quality evaluation was performed using the quality evaluation criteria for randomized controlled trials recommended in the Cochrane Systematic Evaluation Manual, version 5.1.0: (A) method of random assignment; (B) whether allocation was concealed in real time; (C) whether the study subjects, treatment regimen implementers, and outcome measures were blinded; (D) whether the data were complete; (E) whether there was selective reporting of results; and (F) whether there were other biases, including early trial stop and baseline imbalance. A rating of “low risk of bias,” “high risk of bias,” or “uncertain” was used. Methodological quality was evaluated independently by two evaluators, with third-party input and agreement in case of disagreement. When more than 8 studies were included in the analysis, funnel plots were drawn to analyze whether there was publication bias.

### 2.3. Statistical Analyses

Meta-analysis was performed using Rev Man 5.4.1 software. The MD and its 95% CI were used to express the measurement data, and the odds ratio (OR) and its 95% CI were used to express the count data. The studies were tested for heterogeneity, and if there was no heterogeneity or small heterogeneity (*I*^2^ ≤ 50%, *P* > 0.05), a fixed-effects model was used to calculate the combined effect size; conversely, if the heterogeneity was large (*I*^2^ > 50%, *P* < 0.05), the sources and causes of heterogeneity were analyzed; and if there was only statistical heterogeneity, a random-effects model was used to combine the effect sizes; otherwise, only descriptive analysis was performed. *P* < 0.01 indicates that the difference is statistically significant.

## 3. Results

### 3.1. Process and Results of the Included Literature

**Figure 1:**
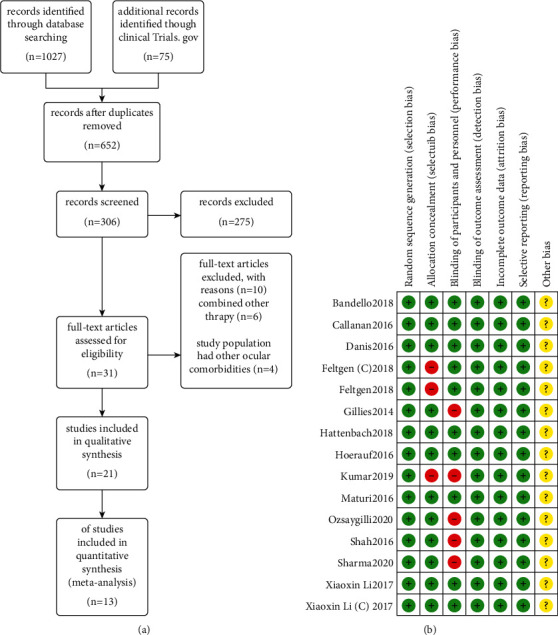
(a) Study flow diagram. (b) Distribution of risk of bias of included RCTs.

The initial screening yielded 1102 relevant articles, and 652 articles were obtained after eliminating duplicates. 306 articles were selected for full-text reading after reading the titles and abstracts, 31 articles were included for analysis according to the nadir criteria, 21 articles were included in the qualitative evaluation, 8 RCTs were excluded due to incomplete data on study index units or observation of final outcome indicators, and finally, only 13 RCTs were included in this study. The specific incorporation and exclusion process is shown in Figure 1(a). The evaluation of the quality of the literature showed that 5 articles were at high risk of “implementation blind bias” and 2 articles were at high risk of “bias.” The “other bias” of 13 articles was not determined, as shown in Figure 1(b).

### 3.2. Summary of Baseline Characteristics of Included Literature Studies

Seven RCTs included populations of patients with DME and six RCTs of patients with RVO-ME. All the included RCTs involved a total of 3206 patients, with 1573 in the trial group and 1633 in the control group. The basic characteristics of the 13 RCTs are shown in Table 1.

## 4. Meta-Analysis Results

### 4.1. Effectiveness Outcomes

#### 4.1.1. BCVA and CRT for DME

**Figure 2:**
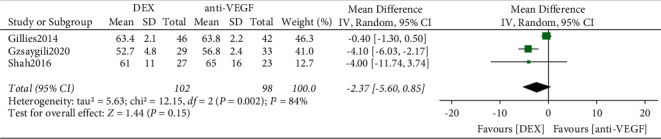
Forest plot of BCVA for DME.

Three RCTs were performed to analyze BCVA improvement rates for DME patients. Heterogeneity among studies in the DME population was statistically significant (*I*^2^ = 84%, *P*=0.002), and the large heterogeneity was considered due to the small number of included studies, so a random-effects model was used to calculate the combined effect size. The results showed that the difference between DEX and anti-VEGF in the BCVA improvement rate was not statistically significant (*P*=0.15) (Figure 2).

**Figure 3:**
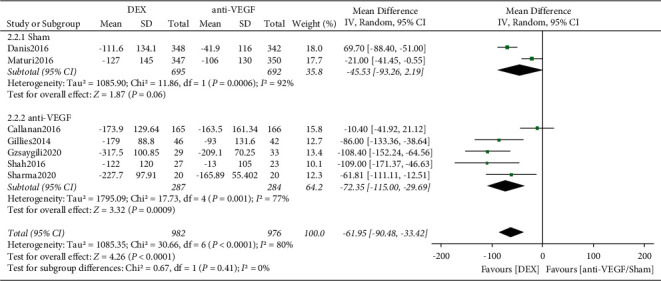
Forest plots of CRT for DME.

A total of 7 RCTs reported data on CRT changes in DME patients, the statistical significance of heterogeneity between studies in the DME population (*I*^*2*^ = 80%, *P* < 0.0001) was observed, and the combined effect size was calculated using a random-effects model. The results showed a statistically significant difference in CRT reduction rates between DEX and anti-VEGF/sham (MD = −61.95, 95% CI: −90.48∼−33.42, *P* < 0.0001). According to the different control groups, subgroup analysis was performed. Subgroup analysis showed that the difference in CRT reduction rate between the DEX and sham groups was not statistically significant (*P*=0.06), and the difference in CRT reduction rate between the DEX and anti-VEGF groups was statistically significant (MD = −72.35, 95% CI: −115.0∼−29.69, *P*=0.0009) (Figure 3). This suggests that different control groups are not the cause of heterogeneity. The obvious clinical heterogeneity may be caused by the small sample size of included studies.

#### 4.1.2. BCVA and CRT for RVO-ME

**Figure 4:**
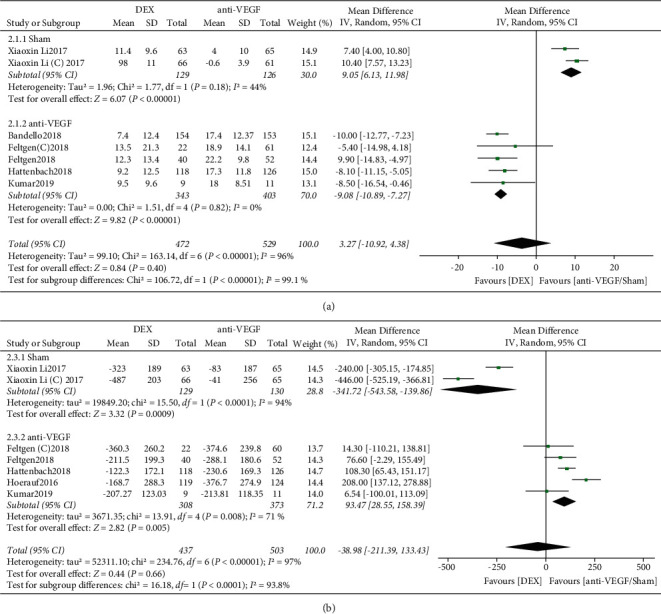
(a) Forest plot of BCVA for RVO-ME. (b) Forest plot of CRT for RVO-ME.

A total of 5 RCTs were performed to analyze BCVA improvement rates, including the study by Feltgen and Xiaoxin Li, which divided patients into 2 subgroups (BRVO and CRVO), and the control group in the study by Xiaoxin Li was all sham injections, whereas in the other studies, the control group was all anti-VEGF treatment. There was statistically significant heterogeneity between studies in the RVO-ME population (*I*^2^ = 96%, *P* < 0.00001), and a random-effects model was used to calculate the combined effect size. The results showed no statistically significant difference between DEX and anti-VEGF/sham in BCVA improvement rates (*P*=0.40). Considering the large heterogeneity of the included literature, a subgroup analysis was performed, with the studies in which the control group was the sham injection group divided into a separate subgroup and the studies in which the control group was treated with anti-VEGF as another subgroup. A random-effects model was used to calculate the combined effect size, and the results showed that there was no statistically significant heterogeneity in the sham group (*I*^2^ = 44%, *P*=0.18), and the difference in BCVA improvement rate between the DEX and sham groups was statistically significant (MD = 9.05, 95%CI:6.13∼11.98, *P* < 0.00001). The heterogeneity in the anti-VEGF group was not statistically significant (*I*^2^ = 0, *P*=0.84), and the difference between DEX and anti-VEGF in BCVA improvement rate was statistically significant (MD = −9.08,95% CI:−10.89∼−7.27, *P* < 0.00001) (Figure 4(a)). Thus, the source of heterogeneity between studies in the RVO-ME population was the sham group in the included studies.

5 RCTs were performed to analyze CRT changes in DME patients. Heterogeneity between studies in the RVO-ME population was statistically significant (*I*^2^ = 97%, *P* < 0.00001), and the combined effect sizes were calculated using random effects. The results showed no statistically significant difference between DEX and anti-VEGF/sham in the rate of CRT reduction (*P*=0.66). Subgroup analysis showed a statistically significant difference in CRT reduction rate between the DEX and sham groups (MD = −341.72,95% CI:−543.58∼−139.86, *P*=0.0009) and a statistically significant difference in CRT reduction rate between the DEX and anti-VEGF groups (MD = 93.47, 95% CI: 28.55–159.39, *P*=0.005) (Figure 4(b)). The obvious clinical heterogeneity may be caused by the small sample size of included studies, or it could be that the study included both CRVO and BRVO patient populations.

### 4.2. Security Endings

#### 4.2.1. Adverse Reactions for DME

**Figure 5:**
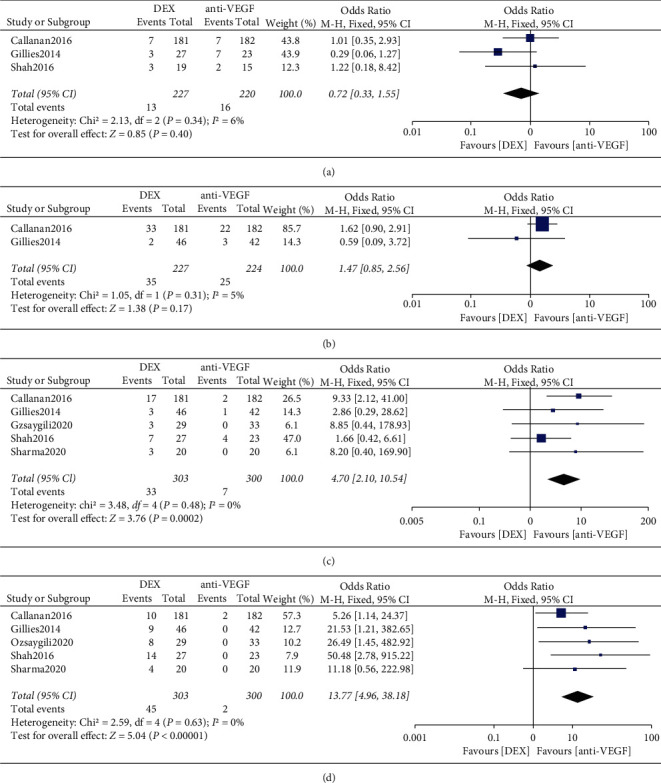
(a) Forest plot of SAEs, (b) conjunctival hemorrhage, (c) cataract or cataract exacerbation, and (d) high IOP for DME.

A total of 3 RCTs were performed to analyze the incidence of SAEs. Heterogeneity between studies in the DME population was not statistically significant (*I*^2^ = 6, *P*=0.34), and a fixed-effects model was used to calculate the combined effect size. There was no statistically significant difference in the incidence of SAEs between DEX and anti-VEGF (*P*=0.40) (Figure 5(a)). 2 RCTs were performed to analyze the incidence of conjunctival hemorrhage. Heterogeneity between studies in the DME population was not statistically significant (*I*^2^ = 5%, *P*=0.31), and the combined effect size was calculated using a fixed-effects model. The results showed that the difference in the incidence of conjunctival hemorrhage between DEX and anti-VEGF was not statistically significant (*P*=0.17) (Figure 5(b)). 5 RCTs were performed to analyze the incidence of cataract or cataract exacerbation. Heterogeneity between studies in the DME population was not statistically significant (*I*^2^ = 0, *P*=0.48), and a fixed-effects model was used to calculate the combined effect size. The results showed a statistically significant difference in the incidence of cataract or cataract exacerbation between DEX and anti-VEGF (10.89% (33/303) versus 2.33% (7/300), OR = 4.70, 95% CI: 2.10 to 10.54, *P*=0.0002*P*=0.0002) (Figure 5(c)). 5 RCTs were analyzed for treatment-induced high IOP. Heterogeneity between studies in the DME population was not statistically significant (*I*^2^ = 0, *P*=0.6), and a fixed-effects model was used to calculate the combined effect size. The results showed a statistically significant difference in the incidence of high IOP between DEX and anti-VEGF (14.85% (45/303) versus 0.67% (2/300), OR = 13.77, 95% CI: 4.96–38.18, *P* < 0.00001*P* < 0.00001) (Figure 5(d)).

#### 4.2.2. Adverse Reactions for RVO-ME

**Figure 6:**
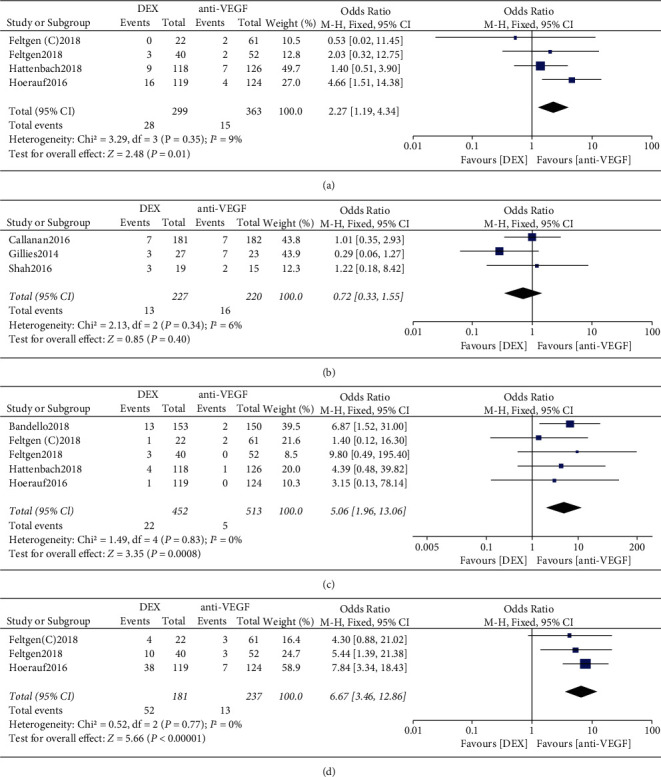
(a) Forest plot of SAEs, (b) conjunctival hemorrhage, (c) cataract or cataract exacerbation, and (d) high IOP for RVO-ME.

There was no statistically significant heterogeneity between studies in the RVO-ME population (*I*^2^ = 9%, *P*=0.35), and a fixed effect was used to calculate the combined effect size. The results showed no statistically significant difference between DEX and anti-VEGF in the incidence of SAEs (*P*=0.01) (Figure 6(a)). Heterogeneity between studies in the RVO-ME population was not statistically significant (*I*^2^ = 6%, *P*=0.34), and a fixed-effects model was used to calculate the combined effect size. The results showed no statistically significant difference in the incidence of conjunctival hemorrhage between DEX and anti-VEGF (*P*=0.40) (Figure 6(b)). There was no statistically significant heterogeneity between studies in the RVO-ME population (*I*^2^ = 0, *P*=0.83), and a fixed-effect model was used to calculate the combined effect size. The results showed a statistically significant difference in the incidence of cataractogenesis or cataract exacerbation between DEX and anti-VEGF (4.87% (22/452) versus 2.97% (5/513), OR = 5.06, 95% CI: 1.96 to 13.06, *P*=0.0008) (Figure 6(c)). Heterogeneity between studies in the RVO-ME population was not statistically significant (*I*^2^ = 0, *P*=0.77), and a fixed-effect model was used to calculate the combined effect size. The results showed a statistically significant difference in the incidence of high IOP between DEX and anti-VEGF (4.87% (52/181) versus 0.97% (13/237), OR = 6.67, 95% CI: 3.46 to 12.86, *P* < 0.00001) (Figure 6(d)).

### 4.3. Sensitivity Analysis

The results of the sensitivity analysis by excluding each study individually showed that the differences between the results after exclusion and before exclusion were not statistically significant (all *P* ≥ 0.1), suggesting that the results of this meta-analysis are more stable and reliable.

## 5. Discussion

This study conducted a meta-analysis of 7 RCTs on DEX in DME and 6 RCTs on DEX in ME secondary to RVO. 13 RCTs included a total of 3206 patients, including 1958 patients with DME, 982 treated with DEX, and 976 controls (including 284 treated with anti-VEGF and 692 in the sham injection group). There were 1248 patients with RVO-ME, 591 cases treated with DEX, and 657 cases in the control group (including 527 cases treated with anti-VEGF and 130 cases in the sham injection group). Our findings showed that both DEX and anti-VEGF treatment significantly improved visual function and retinal macular anatomical morphology during early treatment of ME. In this study, it was shown that there was no difference between DEX and anti-VEGF treatment in the rate of improvement of BCVA in patients with DME and that DEX was more effective than anti-VEGF in terms of CRT reduction but slightly less safe than anti-VEGF treatment. In terms of BCVA improvement and CRT reduction in RVO-ME patients, DEX was more effective than anti-VEGF treatment but slightly less safe than anti-VEGF treatment.

An increasing number of studies have shown [19, 20] that inflammation is strongly associated with early neuromicrovascular pathological changes in DR, that oxidative stress, formation of late glycosylation end products, and increased VEGF expression all exacerbate the intraretinal inflammatory response, and that higher concentrations of proinflammatory cytokines TNF-*α*, IL-1*β*, IL-6, and CAM1 in DR patients induce sustained intraretinal. This leads to increased vascular permeability, BRB dysfunction, and ME formation. In addition, DME has been shown to be associated with elevated inflammatory cytokines such as TNF-*α*, IL-1, IL-10, and IL-12 in atrial or vitreous fluid [21]. The inflammatory response is the key pathogenesis of DME, and early and timely anti-inflammatory treatment is crucial. The results of this study did not differ from anti-VEGF treatment in improving visual outcomes in patients with DME, which is consistent with previous studies [22] that reported no major differences between intravitreal injection of ranibizumab and intravitreal implantation of corticosteroids in improving BCVA in patients with DME; this study showed that DEX treatment was more effective than anti-VEGF treatment in improving the anatomical morphology of the macular retina, which is consistent with the reported data. The present study showed that DEX treatment was superior to anti-VEGF treatment in improving the anatomical morphology of the macular retina, which is different from the study that reported that intravitreal corticosteroid implantation was inferior to ranibizumab in improving the anatomical morphology of the macula. There are two possible reasons for this discrepancy. First of all, there were several different types of anti-VEGF drugs (including the sham injection group) in the control group. In the second place, the sample size included in the previously published literature was small and only one anti-VEGF drug was studied (e.g., razumab) with DEX for comparative analysis [22]. Based on the results of this study and many years of clinical experience of our team, we recommend the timely intervention of DEX with a lower mean number of injections and follow-ups for the treatment of DME, especially in patients with refractory DME with a poor response to anti-VEGF drugs and a high number of relapses. A recent study by Yuan [23] also confirmed that in patients with refractory DME, switching to DEX can significantly improve macular anatomical morphology and visual function. The effectiveness of DEX for treating refractory DME that proved unresponsive to previous anti-VEGF treatments may be attributed to its strong antiangiogenic, anti-inflammatory, and antiedema abilities. In addition to VEGF, other factors such as proinflammatory cytokines may play a significant role in the pathogenesis of refractory DME. The DEX can decrease the expression of VEGF and proinflammatory cytokines, inhibit leukostasis, and reduce vascular leakage [24]. At the same time, the current standard of care could be changed because the use of DEX can improve the treatment effect of macular edema and reduce the number of hospital visits for DME patients.

It is worth noting that optical coherence tomography (OCT) is a key adjunct to the diagnosis and evaluation of drug efficacy. Iglicki et al [25] described a new feature of OCT called outer retinal hyperreflective deposits (ORYDs) to be used as the basis for drug selection and prognosis of DME patients. In addition, ORYDs may be caused by a large number of inflammatory factors. DEX should be selected for anti-inflammatory treatment of DME when ORYDs are found in OCT. Meanwhile, OCT biomarkers may predict functional and anatomical outcomes in DME patients treated with DEX implants. A study [26] has shown that disorganization of retinal inner layers (DRIL) may serve as a robust biomarker in DME treated by DEX implant. The presence of DRIL means that vision is difficult to recover and macular edema is prone to recurrence, but the DEX implant has the potential to ameliorate DRIL.

The possible main pathogenesis of RVO-ME is retinal vein thrombosis, which causes elevated retinal capillary pressure, damaging the functional structure of the BRB, and increased vascular permeability due to excess VEGF and inflammatory cytokines, ultimately leading to ME [27]. The most important goals of clinical treatment of RVO-ME are to improve visual quality, improve central vision and provide timely control and relief of ME to avoid more severe complications. The most important goals of clinical treatment for RVO-ME are to improve visual quality, improve central visual acuity, manage and relieve ME in a timely manner, and avoid irreversible damage to the outer layers of the retina, especially the photoreceptor layer, due to more severe complications of RVO. A past prospective study reported [28] that the difference between DEX and anti-VEGF treatment was not significant in terms of improving BCVA and reducing CRT, and a randomized clinical study [22] showed that anti-VEGF treatment was more effective than DEX in reducing CRT; these controversial studies confirm the effectiveness and necessity of early treatment of RVO-ME with DEX and anti-VEGF. The present study systematically evaluated the efficacy of initial treatment with DEX to improve BCVA and CRT in RVO-ME over not only placebo sham injections but also anti-VEGF agents with a follow-up of 6–12 months.

SAEs include both systemic and ocular localized conditions, with systemic SAEs inducing cardiovascular events, pneumonia, and pyelonephritis , and ocular localized conditions causing iris neovascularization, ocular ischemic syndrome, choroiditis, endophthalmitis, cellulitis, vitreous hemorrhage, and cranial nerve VI palsy. In this study, the safety of DEX and anti-VEGF in the treatment of RVO-ME and DME patients was comparable in terms of SAEs and subconjunctival hemorrhage, and the inferior safety of DEX compared with anti-VEGF was mainly in terms of early cataract development or exacerbation and high intraocular pressure, which is consistent with previous studies reporting that the most common significant ocular side effects of corticosteroids were complications of cataract and increased intraocular pressure [7, 22]. The literature included in this study shows that very few cataracts require surgical intervention, most high IOP is controlled by observation or medication, and very few have to be intervened by laser or surgical procedures.

### 5.1. Quality of Evidence

We evaluated the quality of evidence using the Cochrane Systematic Evaluation Manual, version 5.1.0. The quality of evidence in the overall outcome was relatively high. Both DME patients and RVO-ME patients in the included studies underwent OCT examination and met the clinical diagnostic criteria for DME and RVO-ME. But 5 studies did not implement single-blind or double-blind measures, and 2 studies did not allocate hidden experimental drugs. This would affect the credibility of the evidence quality of this study, though all the studies adopted the randomized number table method, and the data were relatively complete. More adequately powered RCTs are needed in the future.

### 5.2. Potential Limitations of the Study

Certain limitations of this study include the fact that the study did not analyze the duration of each RCT in groups. In addition, we did not specifically classify the control group of anti-VEGF drugs in the study and the unequal DEX dosing intervals among studies, which may have caused some bias. Furthermore, the studies only compared DEX monotherapy and did not analyze studies of combination DEX therapy. Finally, the analysis results of CRT for DME and RVO-ME showed great heterogeneity, and the subgroup analysis failed to solve this problem. Although we analyzed the possible causes of clinical heterogeneity and adopted the random effect model, we did not find out the specific reason which had a certain impact on the accuracy of the results.

## 6. Conclusion

In conclusion, the results of this study show that DEX has better efficacy than anti-VEGF in the DME and RVO-ME populations, but the safety profile is inferior to that of anti-VEGF. However, due to the small sample size and obvious clinical heterogeneity of some research results, more multicenter, large-sample randomized studies are needed to reduce or eliminate adverse effects and to explore the best treatment options for complications and improve the safety of drug use so that the clinical use of DEX can benefit more patients.

## Figures and Tables

**Table 1:** Characteristics of included studies.

The first author	Date	Disease	Intervene measures		BCVA baseline value		CRT baseline value	
			Treatment group	Control group	Treatment group	Control group	Treatment group	Control group
Callanan [6]	2016	DME	On the 1st/5th/10th month, 0.7 mg DEX	PRN after IVR 0.5 mg	60.2 ± 9.74	60.4 ± 9.34	465 ± 136	471 ± 140
Danis [7]	2016	DME	0.7 or 0.35 mg DEX	Sham injection	N/A	N/A	463 ± 156	463.9 ± 132.6
Maturi [8]	2016	DME	0.7 or 0.35 mg DEX and PRN after 6 months	Sham injection	N/A	N/A	469 ± 171	462 ± 152
Ozsaygili [9]	2020	DME	PRN after 0.7 mg DEX	PRN after IVA 2 mg for 3 months	46.3 ± 4.4	47.5 ± 3.1	615.2 ± 150.4	576.5 ± 75.3
Shah [10]	2016	DME	0.7 mg DEX at 1, 3, and 6 months	IVR 1.25 mg for consecutive months	59 ± 13	59 ± 12	485 ± 122	458 ± 100
Sharma [11]	2020	DME	0.7 mg DEX and PRN after 3 months	IVB1.25 mg/IVR 0.5 mg and PRN after 1 month	N/A	N/A	460.95 ± 125.46	443.55 ± 131.53
Gillies [12]	2014	DME	Every four months 0.7 mg DEX and PRN	Every one month IVB 0.5 mg and PRN	55.5 ± 12.5	56.3 ± 11.9	474.3 ± 95.9	503 ± 140.9
Xiaoxin Li [13]	2017	BRVO CRVO	0.7 mg DEX	Sham injection	52.6 ± 10.8	53.1 ± 10.5	683 ± 242	643 ± 213
			0.7 mg DEX	Sham injection				
Bandello [14]	2018	BRVO	PRN after 0.7 mg DEX was given in the 1st and 5th month	Continuous 5 months of IVR and PRN	59.2 ± 10.9	547 ± 163	544 ± 168	59.2 ± 10.9
Feltgen [15]	2018	BRVO	0.7 mg DEX and PRN	0.5 mg IVR and PRN	58.3 ± 10.8	56.8 ± 10.0	547.3 ± 178.9	547.3 ± 178.9
		CRVO	0.7 mg DEX and PRN	0.5 mg IVR and PRN	53.2 ± 16.1	54.1 ± 15.8	721.2 ± 231.1	698.8 ± 228.6
Hattenbach [16]	2018	BRVO	0.7 mg DEX and sham injection after two months and PRN	Continuous 3 months of IVR and PRN	N/A	N/A	N/A	N/A
Hoerauf [17]	2016	CRVO	0.7 mg DEX and sham injection after six months	Continuous 3 months of IVR and PRN	51.5 ± 15.6	51.7 ± 16.5	705.2 ± 231.1	723.8 ± 245.9
Kumar [18]	2019	BRVO	0.7 mg DEX	Continuous 3 months of IVR 0.5 mg	0.64 ± 0.15 (6/24)	0.68 ± 0.13 (6/30)	493.67 ± 100.79	487.53 ± 105.90

IVA: intravitreal aflibercept; IVB: intravitreal bevacizumab; IVR: intravitreal ranibizumab.

## Data Availability

The Cochrane Library, PubMed, Embase, Web of Science, and Clinical Trials. gov were searched until March 2022. Subject search terms were as follows: dexamethasone intravitreal implant; macular edema; retinal vein occlusion; diabetic retinopathy; and randomized controlled trial; and free search terms are listed in the accompanying table.
